# Effective Noise Reduction in NDR Systems: A Simple Yet Powerful Apriori-Based Approach

**DOI:** 10.3390/s24206547

**Published:** 2024-10-11

**Authors:** Sajad Homayoun, Magnea Haraldsdóttir, Emil Lynge, Christian D. Jensen

**Affiliations:** 1 Cyber Security Group, CMI Section, Department of Electronic Systems, Aalborg University, 2450 Copenhagen, Denmark; 2 Muninn Innovation Lab, Muninn ApS, 2800 Kongens Lyngby, Denmark; mha@muninn.ai (M.H.); ely@muninn.ai (E.L.); 3 Department of Electrical and Computer Engineering, Aarhus University, 8200 Aarhus, Denmark; cdj@ece.au.dk

**Keywords:** Network Detection and Response (NDR), security alerts, noise alert filtering, Apriori algorithm

## Abstract

Noise (un-important) alerts are generally considered a major challenge in intrusion detection systems/sensors because they require more analysts to review and may cause disruption to systems that are shut down to avoid the consequences of a compromise. However, in real-world situations, many alerts could be raised for automatic tasks being completed by some software or regular tasks by users doing their daily job. This paper proposes an approach to reduce the number of noise alerts, assuming that frequent long-term security alerts can be considered noise if their frequency is meeting some criteria, such as the minimum occurrence ratio. We prove that to effectively reduce the level of noise alerts in Network Detection and Response (NDR) systems, we are able to use simpler algorithms; sometimes, the answer is in simpler solutions, and not always in complex solutions. We study data from a real customer of a Danish NDR solution and propose an Apriori-based approach to find frequent noisy alerts. Our comparison of the detected noise before and after applying our solution shows high performance in reducing noise alerts for most of the alert types for a real customer. Our experiments show that our method can filter more than 40% of the alerts by setting the minimum occurrences to 70%. Moreover, our results show that we were able to filter out more than 90% for some alert categories.

## 1. Introduction

Alert fatigue is a serious challenge for security analysts. Based on a qualitative study of SOC analysts’ perspectives on security alerts from the University of Oxford, most alerts are triggered by legitimate behavior in the organization’s environment, which analysts may choose to ignore [1]. Most existing research aimed at reducing the rate of noise alerts has focused on proposing new methods for detecting attacks and intrusions. However, fewer studies consider security alerts as their input at a higher level. This higher-order analysis involves examining alerts that are the outputs of intrusion detection systems, which are directly extracted from raw data such as network traffic or system logs. Therefore, this paper presents a solution designed to assist security analysts in reducing potential noise alerts by suggesting several filtering rules.

Current approaches often center on minimizing false positives and utilizing predefined rule-based alert correlations. While these methods are crucial, they tend to overlook the repetitive nature of certain alerts. These repetitive alerts, although individually unimportant, can accumulate to overwhelm the system. This flood of alerts can lead to alert fatigue, where security analysts become desensitized to alerts, potentially missing genuine threats amid the noise. Furthermore, the sheer volume of repetitive alerts can obscure significant threats, making it challenging to identify and respond to critical security incidents promptly. Our proposed method addresses this gap by focusing on the reduction of repetitive, unimportant alerts, thereby enhancing the efficiency and effectiveness of the alert management process in security operations centers (SOCs).

- **A new perspective on security alerts analysis using “market basket analysis”:** This research introduces a fresh approach to analyzing security alerts by applying the principles of market basket analysis. While market basket analysis itself is a well-known technique, using it to detect repetitive alerts in security datasets might be a new twist. By treating each alert as if it were a shopping basket, we can spot patterns and frequent items within these baskets, leading to more insightful analysis. This approach moves beyond traditional alert analysis methods, giving us a clearer picture of alert content. This can help reduce noise and make security operations more efficient. Even if this application is not entirely unprecedented, our approach offers a fresh and potentially impactful perspective that can significantly benefit security analysts.
- **An Apriori-based algorithm for filtering repetitive alerts:** We developed an algorithm based on the Apriori principle, commonly used in market basket analysis, to identify frequent itemsets of alerts. The algorithm suggests filtering rules that help in recognizing and suppressing repetitive, unimportant alerts. This method not only reduces the volume of alerts that security analysts need to review but also enhances their ability to focus on significant threats, thereby reducing the likelihood of alert fatigue and missed detections.
- **A robust performance comparison to detect potential noise alerts:** The paper includes a thorough performance evaluation of the proposed method. By demonstrating the effectiveness of our algorithm in various scenarios and datasets, we provide strong evidence of its capability to detect and filter noise alerts. This robust performance comparison underscores the practical applicability of our method in real-world security operations centers (SOCs), highlighting its potential to significantly improve alert management processes.

It is important to emphasize that our approach goes beyond simply manipulating the presentation of data. The definition of false positives can vary across different enterprises. However, we specifically target repetitive alerts because we believe that they could be a significant cause of alert fatigue. While alerts are mostly raised based on factual events (mostly on signature-based intrusion detection systems), such as user activities, making them not necessarily false, repetitive alerts can often be ignored without missing critical information. By applying market basket analysis, we provide a method to identify and filter out these repetitive alerts based on their content and frequency. This involves analyzing the actual data within each alert, rather than just how the alerts are displayed.

This paper is divided as follows: Section 2 gives an overview of the related works and defines the research gap. Section 3 describes our data collection pipeline, and Section 4 presents our proposed method. We will discuss the experimental results in Section 5, and Section 6 concludes the paper.

## 2. Related Work

Research on NIDS/NDR has extensively explored various methods to enhance security and efficiency. Much of the existing work focuses on the intrusion detection mechanisms, alert correlation, reducing false positives, and applying machine learning techniques to improve the detection ratio [2,3,4]. These efforts have undoubtedly advanced the field, providing robust mechanisms to detect and respond to potential threats. However, there remains a significant gap in the effective identification and filtering of repetitive alerts deemed unimportant.

Current approaches mainly aim to reduce false positives and use predefined rule-based correlations. While effective, they often neglect repetitive alerts, which can flood the system and cause alert fatigue. This desensitization risks missing real threats, and the high volume of these alerts can obscure significant security incidents.

For example, Negi et al. [5] employed context-aware alert correlation using ArcSight SIEM and open-source NIDS, which significantly reduced non-relevant alerts through ontology-based verification. Similarly, Chuvakin et al. [6] discussed comprehensive strategies for logging and log management, emphasizing the importance of efficient alert handling to reduce false positives and enhance security monitoring. However, these studies do not specifically address the issue of repetitive alerts.

Park and Ahn [7] highlighted the role of adaptive models in improving alert filtering accuracy by comparing Snort [8] and Suricata [9] environments. Their work underscores the need for systems that can learn and adapt to new data and dynamic conditions, yet the focus remains on reducing false positives rather than identifying and filtering repetitive alerts.

Moreover, studies like those by Alazzam et al. [10] and Ferrag et al. [11] have proposed innovative feature selection algorithms and hybrid systems combining rule-based and machine learning techniques. While these approaches improve overall detection capabilities, they do not specifically tackle the problem of alert repetitiveness.

The practical implementation of NIDS often involves extensive rule tuning and management, as emphasized by Corelight [12]. Proactive tuning and rule management can prevent alert fatigue and ensure that only high-value alerts are prioritized. However, the reactive nature of this process means it often addresses issues on a case-by-case basis rather than systematically identifying and filtering repetitive alerts.

Given these limitations, our research aims to fill this critical gap by introducing the application of the Apriori algorithm [13] for detecting and filtering unimportant repetitive alerts in NIDS. The Apriori algorithm, traditionally used in market basket analysis, identifies frequent itemsets and association rules. Recent studies have also highlighted its application in diverse domains such as network intrusion detection [14]. By adapting this algorithm to NIDS alert data, we can systematically uncover patterns of repetitive alerts, enabling more efficient filtering processes. This approach not only reduces the noise in the alert stream but also enhances the focus on significant alerts that require immediate attention.

In fact, previous work using the Apriori algorithm to reduce noise in optical networks has been conducted [15]. Wang et al. aim to reduce alerts in optical networks by identifying correlated alerts. This is accomplished by extracting features from the alerts and the algorithm applied to those features. This generates so-called high-frequency chain alerts, and these are then considered the most important in the scope of the paper. This approach showed promising results in noise reduction. In this paper, the frequent itemsets that the algorithm identifies are then considered noise and using these itemsets rules can be proposed. This differs from [15] as, in this case, frequent itemsets will be considered noise and only those alerts that are not highly correlated with other alerts will be considered important.

In summary, while previous studies have laid the groundwork for alert correlation and reduction, our research addresses the overlooked issue of repetitive alerts. By employing the Apriori algorithm, we propose a solution that optimizes the performance and accuracy of NIDS, ensuring that significant threats are not obscured by a flood of unimportant alerts.

## 3. Data Sources and Collection

We used the data from Muninn NDR solution (https://muninn.ai/, accessed on 28 June 2024), which is a Danish security solution with a wide range of customers in Denmark and other European countries from public healthcare to private manufacturing sectors. The dataset is private to one of the customers, and our research team was given access based on explicit customer consent.

### 3.1. Security Alerts Structure

To keep our proposed method applicable to all types of network security alerts, we focus on common meta-data found in most network intrusion detection systems. We define the structure of our alerts as follows:

- Alert Timestamp,
- Source IP,
- Destination IP (if applicable),
- Description,

where the *Description* is a long string generated based on hard-coded scripts in Zeek [16]. This description gives more information about the main cause of the alert. For example, a description for the *ARP scan detected* alert shows the MAC address. Therefore, the description is an important field, and we need to consider that to find frequent alerts.

### 3.2. Data Extraction

We extracted all security alerts generated by Muninn AI Detect, which is an NDR solution, from 1 November 2023 to 31 May 2024. In total, we found approximately ≈59 K alerts for all the alert categories in the studied period. To protect the anonymity of our customer, we are not disclosing the exact number of alerts. This precaution ensures that no one can query the database to identify the specific customer we have been studying.

### 3.3. Initial Analysis

**Figure 1:**
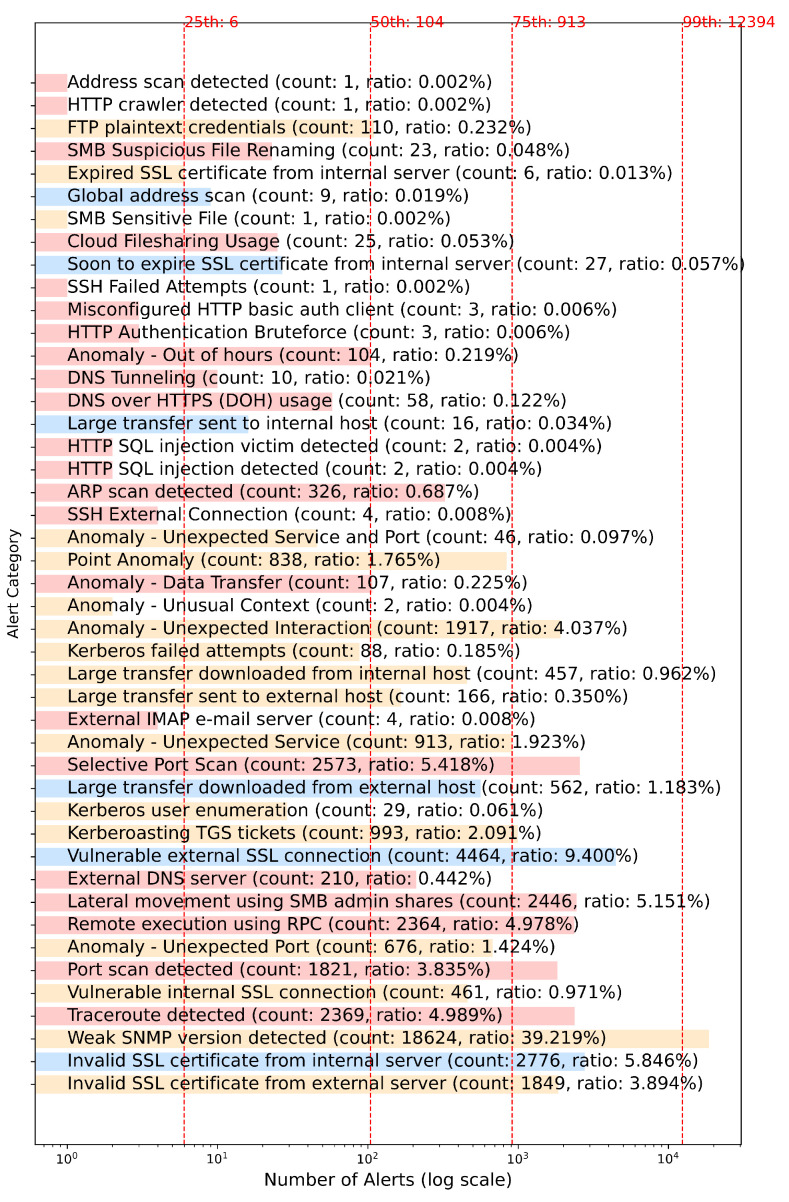
Number of alerts in $D_{1}$ dataset per alert type.

We found 46 unique alerts categories shown in Figure 1 with their frequencies. By looking at the percentiles, we can see that some alert categories are more frequent than the others. The most frequent in Figure 1 is *Weak SNMP version detected*, and it has been seen about 39% of the whole dataset, while there are three alert categories with the least number of alerts (0.002% each). It is obvious from Figure 1 that we could ignore categories with a low frequency of alerts, as one may assume that they will have less impact on security analysts’ feelings of alert fatigue. The figure shows different percentiles, and the proposed method in this paper could be used on alerts of categories meeting a percentile value.

## 4. Method

**Figure 2:**
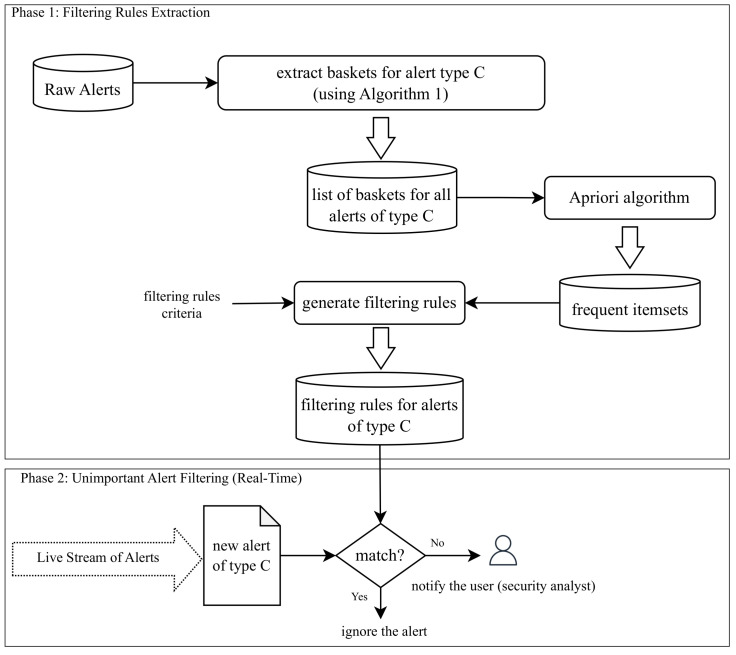
The proposed method for creating alert filtering rules for repetitive alerts.

Market basket analysis has been effectively used in many cases from retail sales optimization to healthcare diagnostics [17], identifying frequent co-occurrences of products or symptoms to enhance decision-making and strategic planning. We believe that using market basket analysis techniques could also be useful in finding repetitive security alerts. Therefore, nothing prevents us from creating one shopping basket per security alert and applying frequent itemset detection algorithms to find the repetitive alerts that could be good candidates for reducing alert fatigue. Figure 2 describes the procedure to extract filtering rules for each alert type using a block diagram, where Phase 1 extracts filtering rules for each alert type *C* using Algorithm 1, and Phase 2 applies the extracted rules to a stream of raw alerts to avoid notifying users when facing unimportant repetitive alerts.
**Algorithm 1** Basket Extraction from Security Alerts**Require:** Data: raw_alerts_collection **Ensure:** List of Baskets: list_of_baskets
 1:list_of_baskets← empty list 2:**for** each raw_alert in raw_alerts_collection **do** 3:      alert_basket← empty unordered list 4:      F← Extract the fundamental items from the raw_alert 5:      alert_basket.add(F) 6:      E← Extract the extended items from the raw_alert based on the predefined patterns for the alert type 7:      alert_basket.add(E) 8:      Add alert_basket to list_of_baskets 9:**end for**10:**return** list_of_baskets


### 4.1. Phase 1: Filtering Rules Extraction

A raw alert follows the structure mentioned in Section 3.1, and we will have a list of baskets for all alerts of the same type in a dataset.

#### 4.1.1. From Raw Alerts Collection to List of Baskets

A basket is an unordered list, where the items do not follow any order in the list. A basket may contain items such as the following:

- Alert source IP address;
- Alert destination IP address (if applicable);
- Alert hour of day in range of 0–23;
- Alert description-specific items.

**Figure 3:**
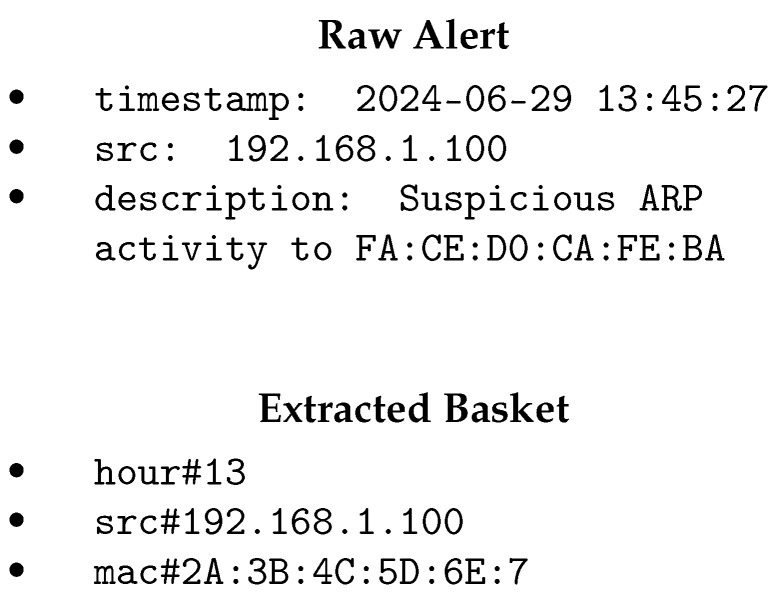
An example of conversion of a raw alert to a basket.

A basket is a an unordered list of items being seen together in an alert, so an alert could be converted to a basket based on the values of its fields. The extracted basket represents the structured attributes derived from the main alerts. Each attribute in the extracted basket corresponds to a specific feature extracted from the main alerts. This structured representation facilitates the further processing and analysis of security events. Figure 3 shows an example of an NDR alert mapped to a market basket. Note that *ARP scan detected* alerts do not have a destination IP address included in the main alert. If the destination exists in the main alert, the extracted basket includes an item *dest#IP_ADDRESS*. We call items extracted directly from the data fields of a raw alert *fundamental alerts*, while any items extracted from the description field of a raw alert would be called *extended items*. In the given example, the timestamp *2024-06-29 13:45:27* from the raw alert has been used to add a *fundamental item* to the basket showing the time of the day of the alert (in this alert, it is 13), while extracting the MAC address from the description field of an alert makes an *extended item* in our alert basket. It is obvious that the structure of baskets could be defined based on a decision made by the user as they want to have filtering rules. For example, a user may want to add the day of the week as an item to their baskets, which would be *day_of_week#Monday* for the given timestamp.

The important note is that we concatenate *field name* and *field value* with a “#” to create the items in a basket. We do this to be able to differentiate source IPs and destination IPs in the baskets and avoid confusion in the filtering rules extraction step and when suggesting filtering rules to the user. For example, source IP and destination IP addresses with “*src#192.168.1.100*” and “*dest#192.168.1.100*” are totally different because security alerts are asymmetric in terms of source and destination addresses. For the alert description, we add the meta-data name. For example, the MAC address in an *ARP scan detected* alert would be “*mac#2A:3B:4C:5D:6E:7*” (see Figure 3).

Algorithm 1 extracts a shopping basket per alert for each alert type. We assume that *raw_alerts_collection* only contains the raw alerts of a single alert type (e.g., *ARP scan detected*). We may run the algorithm for all alert types with alerts counted to be more than a determined percentile (for example, the 50-th percentile).

Below, we provide a detailed explanation of each step in the algorithm:

- Line 1: Initializes *list_of_baskets* as an empty list to store the transformed baskets (alerts turned into unordered lists of items (features));
- Line 2: Iterates over each alert in the *raw_alerts_collection*, ensuring that each alert is processed individually;
- Line 3: Initializes an empty alert basket by creating an unordered list;
- Line 4: Extracts the alert’s fundamental features into *F*, such as the source IP, destination IP, and hour of day, providing the fundamental attributes of the alert. Now, *F* contains the fundamental items like src#192.168.1.100, etc.;
- Line 5: Appends the fundamental items *F* to the current alert basket;
- Line 6: Extracts extended features into *E* from the current raw alert based on predefined patterns for the alert type, such as additional descriptive details from the alert *Description*. Now, *E* contains the extended items like mac#FA:CE:D0:CA:FE:BA etc.;
- Line 7: Appends the extended items *E* to the current alert basket;
- Line 8: Adds the created basket to *list_of_baskets*, effectively storing the transformed alert for future analysis (Apriori algorithm).

Note that we run the algorithm separately on the collection of alerts with the same alert type. Therefore, we will have *Nlist_of_baskets* for a dataset with *N* unique alert types.

#### 4.1.2. Generate Filtering Rules

As Apriori outputs a set of frequent itemsets based on the determined minimum support ratio, we should decide which candidate itemsets meet our required criteria to be added to the collection of filtering rules. This collection of filtering rules will be suggested to the user and finally be used to ignore unimportant repetitive alerts. The important point is that we need have some criteria to suggest the rules to the user (or use them for filtering). Our suggested filtering rules (itemsets) should be specific enough to avoid filtering important alerts. The rules should not be too generic to cover many relevant alerts. It is worth noting that the term “rules” should not be mistaken for association rules. In fact, we can create filtering rules using the frequent itemsets. The following requirements are listed by our security experts:

- All suggested itemsets must include at least one IP address (either source or destination);
- All suggested itemsets must specify an alert type type. We implicitly meet this criterion as we run our method on the data of the same alert type.

### 4.2. Phase 2: Unimportant Alert Filtering (Real-Time)

In this phase, the focus shifts to filtering out unimportant alerts in real-time based on the output from Phase 2. The input is a live stream of alerts, and as each new alert of type C is seen, it is immediately compared against the previously established filtering rules from Phase 1 (for alerts of type C). If the new alert matches any of these filtering rules, it is deemed unimportant and subsequently ignored. This reduces the volume of alerts that need to be processed and reviewed by security analysts, thereby minimizing alert fatigue. However, if the alert does not match any of the filtering rules, it is considered significant, and the user (security analyst) is notified promptly. This ensures that only pertinent alerts are brought to the analyst’s attention, enhancing their ability to focus on genuine threats and improving the overall efficiency of the security operations center. As an example, let us assume we have the filtering rule (*src#192.168.0.102*, *mac#2A:3B:4C:5D:6E:7F*) for *ARP scan detected*, which means that if a new *ARP scan detected* alert has a source IP of 192.168.0.102, and the description contains the specified MAC address, the system will ignore this alert and will not pass it to the user. It is worth noting that the user has full control of the filtering rules by determining the criteria mentioned in Section 4.1.2.

## 5. Experiment Results and Discussion

In this section, we used the dataset from Table 1 to conduct our experiments and report the achieved performance. First, we used $D_{1}$ and followed Phase 1 of Figure 2 to extract filtering rules for each type of alert. We did this for alert types with a number of alerts greater than the 50-th percentile shown in Figure 1. We then extracted the frequent itemsets for each alert type and, next, extracted the filtering rules based on the criteria mentioned in Section 4.1.2. We followed Phase 2 of Figure 2 on $D_{1}$ and $D_{2}$ separately. In other words, we treated the dataset as a live stream and followed Phase 2 to filter out matching alerts with the extracted rules from Phase 1 on $D_{1}$.

We would like to highlight that, for a collection of itemsets extracted with a low minimum support (in the Apriori algorithm), it is very likely that we will have more itemsets meeting the minimum required support, and this means more filtering rules and, consequently, filtering out more alerts. Table 2 shows a few of the extracted itemsets for some alert categories. The first row shows a frequent itemset extracted from all alerts with the *ARP scan detected* alert type. This itemset says that 80% of all *ARP scan detected* alerts are from a machine with the source IP *192.168.0.102* doing an ARP scan for the MAC address *2A:3B:4C:5D:6E:7F*. Row 2 of the table shows that 45% of all *Anomaly—Data Transfer* alerts come from the activities of a machine with the source IP address *192.168.10.20* between 10 p.m. and 11 p.m. (hour 22 of the day). Note that the filtering rule shown in row 4 is not a valid rule as it does not meet our defined *filtering rules criteria* mentioned in Section 4.1.2 because it does not include an IP address.

**Figure 4:**
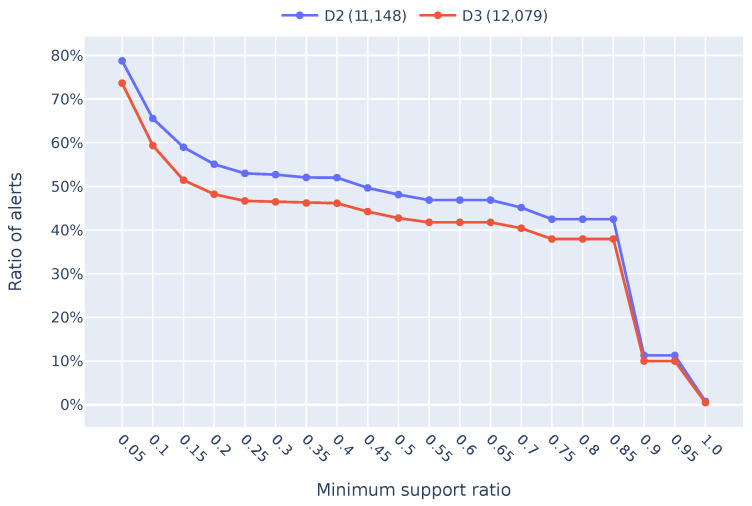
Ratio of unimportant alerts detected by minimum support for all alert categories for $D_{1}$ and $D_{2}$ datasets.

Figure 4 shows how many alerts could be considered potential noise for the datasets $D_{2}$ and $D_{3}$ from Table 1. Essentially, it is about finding the repetitive alerts that can be safely ignored to reduce the burden on security analysts. The graph plots the minimum support ratio on the *x*-axis against the percentage of alerts classified as potential noise/unimportant alerts on the *y*-axis. For instance, when the minimum support ratio is set to 0.05, about 80% of the alerts are identified as potentially unimportant. This means that if an alert appears frequently enough (in this case, at least 5% of the time), it is likely to be unimportant and, potentially, it is noise. Adjusting the minimum support ratio is crucial because it determines the threshold at which alerts are considered repetitive. Our security analyst suggests a threshold of 0.7, meaning that an alert must appear at least 70% of the time to be flagged as noise. This significantly reduces the number of alerts considered unimportant, allowing analysts to focus on more critical issues.

What is interesting in Figure 4 is how the two lines (red for $D_{3}$ and blue for $D_{2}$) almost mirror each other. This consistency shows that the method we used to identify these frequent itemsets is robust and reliable across different datasets. In other words, the frequent itemsets we found in the $D_{1}$ dataset are still relevant and effective when applied to the $D_{3}$ dataset from the next 30 days compared with the $D_{2}$ dataset, which contains data from the last 30 days, even though $D_{3}$ represents new, unseen data. This tells us that our approach works well over time and can enable generalization across different periods. Additionally, it is important to note that we run our analysis separately for each alert type. This means we first identify frequent itemsets within each alert type and then calculate the overall percentage of alerts considered noise by combining the results from all categories. This gives a more accurate and comprehensive picture of how much noise we can filter out.

**Figure 5:**
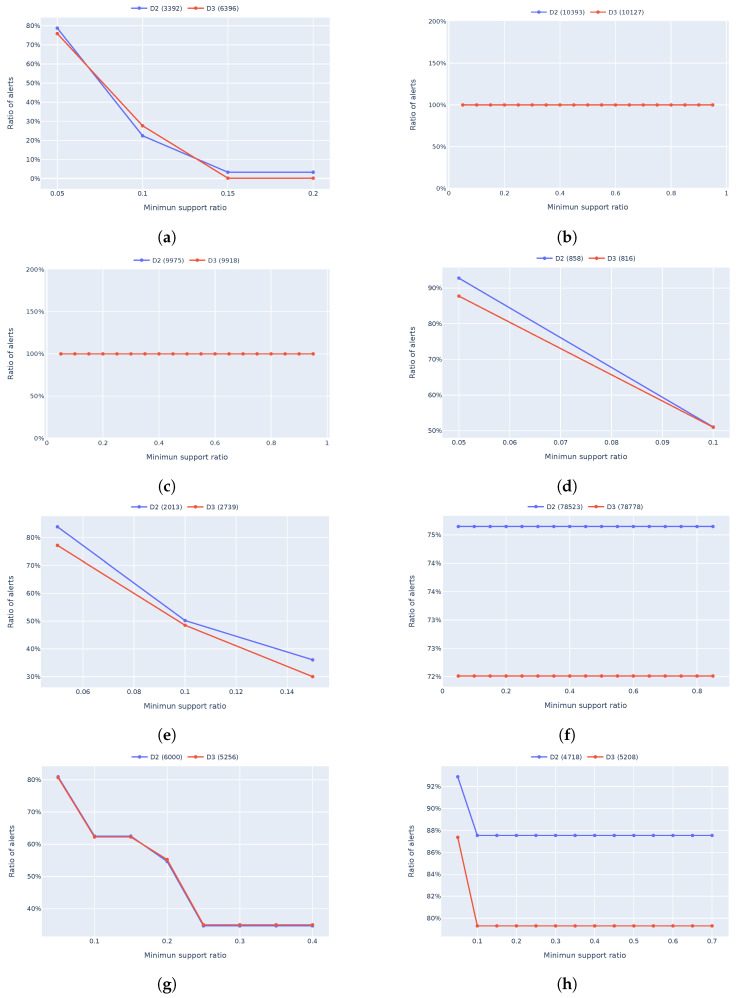
Ratio of unimportant alerts found based on the minimum support for various alert types. (**a**) Vulnerable external SSL connection. (**b**) Lateral movement using SMB admin shares. (**c**) Remote execution using RPC. (**d**) Port scan detected. (**e**) Traceroute detected. (**f**) Weak SNMP version detected. (**g**) Invalid SSL certificate from internal server. (**h**) Invalid SSL certificate from external server.

In addition to the overall comparison (as shown in Figure 4), we have included individual plots for some alert types. To keep the paper concise, we present only a selection of representative plots in Figure 5, which compares the number of alerts identified as potentially unimportant alerts described per alert type. These detailed plots are essential because different alert types may have varying levels of repetitiveness and significance. By examining each alert type separately, we provide a comprehensive analysis that highlights the robustness and adaptability of our approach.

The plots in Figure 5 show that by increasing the minimum support, we flag less alerts as unimportant, and this is because we will have less frequent itemsets once we increase the required minimum support. For a few alert types, we can see a 100% alert reduction and, after checking the datasets and the itemsets, we noted that all alerts of that type are raised for a particular IP address, and the same IP address is being seen in all frequent itemsets leading to a 100% cut in the number of alerts (see Figure 5c as an example). We also investigated the reason for straight lines in the plots of some alert types (e.g., *Weak SNMP version detection*), and we noticed that the main cause is the high frequency (support) of the alerts with that type. In this case, we observed that the majority of alerts (approximately 70–75%) share common values (such as IP addresses). As a result, we identified the same frequent itemsets across all examined minimum support ratios (0.05 to 0.95), leading to consistent filtering rules and the same noise reduction ratio.

Although our performance evaluation only covered two months of data ($D_{2}$ and $D_{3}$), we had a significant volume of alerts during this period, with 11,000 alerts recorded in $D_{2}$ and 12,000 in $D_{3}$ (see Table 1). To evaluate our model effectively, we treated $D_{3}$ as unseen data. This approach helped us determine if the filtering rules we extracted from the seen data in $D_{1}$ could still apply to future, unseen data. For a thorough evaluation, we compared our method’s performance on $D_{2}$, which contains data from the last month of $D_{1}$, with its performance on $D_{3}$. This comparison over a 30-day period allowed us to see how well our extracted filtering rules on $D_{1}$ could filter alerts in a time-based scenario. Essentially, we wanted to ensure that our method could handle both historical and future data effectively, providing a robust solution for managing repetitive alerts.

We would like to mention that there are some limitations to our study in this paper. One limitation of our approach is the underlying assumption that frequent alerts are less important, which might not always hold true across different contexts and domains. While this assumption can help in identifying potential noise, it risks oversimplifying the complexity of alert significance. In certain scenarios, frequent alerts might indicate persistent, critical issues that require immediate attention, such as repeated security breaches or consistent system failures. Therefore, relying solely on frequency to determine the importance of alerts could lead to the unintended consequence of filtering out genuinely significant warnings. To mitigate this risk, it is essential to complement the frequency-based approach with domain-specific knowledge, historical analysis, and expert input, ensuring that critical alerts are not overlooked.

Focusing on the data of one customer could be considered another limitation. The results of our study depend on the behavior of users and their network. In other words, another customer may have a different set of alert categories as Muninn NDR is able to raise more alert categories based on the users’ and network assets’ activities.

It is worth noting that the Muninn NDR solution protects the network by investigating the enterprise networks for 100+ types of alerts, while, for the studied customer, we found only the list shown in Figure 1. This is due to the user and network asset behavior of the studied customer.

## 6. Conclusions

We investigated the possibility of using frequent itemsets to identify repetitive alerts as unimportant alerts. This is based on an assumption that repetitive alerts could be good candidates for being filtered as they could be due to system automation procedures such as backup, system updates, etc. We used data from a real-world dataset of alerts generated by the Muninn NDR solution for a real customer. To extract frequent alert meta-data, we looked at each alert as a shopping basket with items like IP addresses and the time of the day. Then, we used frequent itemset extraction algorithms like Apriori to have a list of items being seen enough times together. Our results show that, based on the level of sensitivity (minimum support) determined by the user, we are able to reduce the number of alerts to be investigated by security analysts. The results shows that by increasing the sensitivity, less alerts will be covered as unimportant, while we will have more frequent itemsets. Our results show that by setting the minimum support to 0.7, there is a potential of flagging around 40% of alerts as unimportant or repetitive, which means a significant reduction in the workload of the human security analysts. Based on the type of the alerts, we might be able to filter out more than 90% of alerts, as they are repetitive for a single host conducting an activity such as backing up servers.

## Figures and Tables

**Table 1:** Dataset description.

Name	From	To	No. of Alerts
$D_{1}$	1 November 2023	31 March 2024	≈36,000
$D_{2}$	1 March 2024	31 March 2024	≈11,000
$D_{3}$	1 April 2024	30 April 2024	≈12,000

**Table 2:** A few of the extracted frequent itemsets (IP/MAC addresses are fictitious to protect the studied customer’s privacy).

No.	Alert Type	Frequent Itemset	Sup. Ratio	Valid Rule
1	*ARP scan detected*	(*src#192.168.0.102*, *mac#2A:3B:4C:5D:6E:7F*)	0.8	✓
2	*Anomaly—Data Transfer*	(*src#192.168.10.20*, *hour#22*)	0.45	✓
3	*External DNS server*	(*src#192.168.0.100*, *server#8.8.8.8*, *hour#9*)	0.68	✓
4	*External DNS server*	(*server#8.8.8.8*)	0.9	×

## Data Availability

The data supporting the findings of this study are not publicly available due to confidentiality and privacy restrictions. The data consist of customer security-related information and security alerts, which are highly confidential and cannot be shared.
